# Effects of acute versus repeated cocaine exposure on the expression of endocannabinoid signaling-related proteins in the mouse cerebellum

**DOI:** 10.3389/fnint.2014.00022

**Published:** 2014-03-05

**Authors:** Ana Palomino, Francisco-Javier Pavón, Eduardo Blanco-Calvo, Antonia Serrano, Sergio Arrabal, Patricia Rivera, Francisco Alén, Antonio Vargas, Ainhoa Bilbao, Leticia Rubio, Fernando Rodríguez de Fonseca, Juan Suárez

**Affiliations:** ^1^Laboratorio de Investigación (Unidad de Gestión Clínica de Salud Mental), Instituto de Investigación Biomédica de Málaga, Hospital Regional Universitario de MálagaMálaga, Spain; ^2^Departament de Pedagogia i Psicologia, Facultat de Ciències de l’Educació, Universitat de LleidaLleida, Spain; ^3^Departamento de Psicobiología, Facultad de Psicología, Universidad ComplutenseMadrid, Spain; ^4^Institute of Psychopharmacology, Central Institute of Mental Health, Medical Faculty of Mannheim, University of HeidelbergMannheim, Germany; ^5^Departamento de Anatomía y Medicina Legal y Forense, Facultad de Medicina, Universidad de MálagaMálaga, Spain

**Keywords:** cocaine, sensitization, cannabinoid, glutamate, tyrosine hydroxylase, mouse, cerebellum

## Abstract

Growing awareness of cerebellar involvement in addiction is based on the cerebellum’s intermediary position between motor and reward, potentially acting as an interface between motivational and cognitive functions. Here, we examined the impact of acute and repeated cocaine exposure on the two main signaling systems in the mouse cerebellum: the endocannabinoid (eCB) and glutamate systems. To this end, we investigated whether eCB signaling-related gene and protein expression {cannabinoid receptor type 1 receptors and enzymes that produce [diacylglycerol lipase alpha/beta (DAGLα/β) and *N*-acyl phosphatidylethanolamine phospholipase D (NAPE-PLD)] and degrade [monoacylglycerol lipase (MAGL) and fatty acid amino hydrolase (FAAH)] eCB} were altered. In addition, we analyzed the gene expression of relevant components of the glutamate signaling system [glutamate synthesizing enzymes liver-type glutaminase isoform (LGA) and kidney-type glutaminase isoform (KGA), metabotropic glutamatergic receptor (mGluR3/5), NMDA-ionotropic glutamatergic receptor (NR1/2A/2B/2C) and AMPA-ionotropic receptor subunits (GluR1/2/3/4)] and the gene expression of tyrosine hydroxylase (TH), the rate-limiting enzyme in catecholamine biosynthesis, because noradrenergic terminals innervate the cerebellar cortex. Results indicated that acute cocaine exposure decreased DAGLα expression, suggesting a down-regulation of 2-arachidonylglycerol (2-AG) production, as well as gene expression of TH, KGA, mGluR3 and all ionotropic receptor subunits analyzed in the cerebellum. The acquisition of conditioned locomotion and sensitization after repeated cocaine exposure were associated with an increased NAPE-PLD/FAAH ratio, suggesting enhanced anandamide production, and a decreased DAGLβ/MAGL ratio, suggesting decreased 2-AG generation. Repeated cocaine also increased LGA gene expression but had no effect on glutamate receptors. These findings indicate that acute cocaine modulates the expression of the eCB and glutamate systems. Repeated cocaine results in normalization of glutamate receptor expression, although sustained changes in eCB is observed. We suggest that cocaine-induced alterations to cerebellar eCB should be considered when analyzing the adaptations imposed by psychostimulants that lead to addiction.

## INTRODUCTION

The neurobiological mechanisms underlying the formation, maintenance and retrieval of drug-related behaviors have been associated with a neuroanatomical circuit to which the contribution of the cerebellum is not considered. Reward-associated conditioned behaviors are controlled by dopamine (DA)-glutamate interactions in regions of the mesolimbic pathway, such as the ventral tegmental area (VTA), nucleus accumbens (NAc), prefrontal cortex (PFCx), dorsal striatum (Str), hippocampus (Hc) and others (Bower and Parsons, 2003). Neuromodulatory systems, such as the endocannabinoid (eCB) signaling system, participate in the control of the DA-glutamate interactions that sustain drug-associated conditioning. Thus, the presence of eCB in these brain pathways has been implicated in the neurochemical and behavioral effects of psychostimulants (Gorriti et al., 1999; De Vries and Schoffelmeer, 2005; Maldonado et al., 2006). Endocannabinoid production is triggered by the activation of DA D2 receptors (Giuffrida et al., 1999). These endocannabinoids modulate synaptic plasticity through stimulation of cannabinoid receptor type 1 (CB1) receptors located in the PFCx glutamatergic axon terminals that innervate the Str (Gerdeman et al., 2002; Freund et al., 2003; Piomelli, 2003; Ronesi and Lovinger, 2005; Mathur et al., 2013). Chronic drug abuse produces a re-organization (neuroadaptation) of the prefronto-striatal-limbic network via its effect on neurotransmitter and neuromodulator systems and their functional interactions (Nestler, 2005; Noori et al., 2012). Outside this circuit, the cerebellum has a relevant catecholaminergic innervation that modulates glutamatergic transmission. However, there is no study addressing the interactions between these two signaling systems in the cerebellum in the context of addiction.

It is becoming clear that the cerebellum plays a potential intermediary role between different brain areas involved in drug-related behavior, such as the prefrontal and associative non-motor cortices, the basal ganglia and the limbic system (Heath et al., 1978; Bostan and Strick, 2010). Moreover, the medial part of the cerebellum has bidirectional connections with dopaminergic VTA neurons (Schweighofer et al., 2004). All of these anatomical findings challenge the traditional view of the cerebellum and support its involvement in functional networks affected by drug addiction (Miquel et al., 2009).

Considering both the anatomical relationship of the cerebellum with major brain pathways involved in drug addiction and the existence of a network of noradrenergic projections to the cerebellar cortex on which cocaine might act, it is reasonable to think that acute/chronic cocaine use might have an impact on the main cerebellar transmission systems. Previous works have demonstrated that cocaine modulates not only dopaminergic activity but also norepinephrine- and serotonin-mediated actions in the brain (Pitts and Marwah, 1987). Thus, the action of cocaine in noradrenergic target circuits could also contribute to the behavioral response. Elevated catecholamine transmission is a primary mediator of cocaine addiction, and repeated exposure to cocaine is associated with the recruitment of glutaminase expression and glutamatergic transmission (Glowinski and Axelrod, 1965; Cornish and Kalivas, 2000; Malenka and Bear, 2004; Lopez-Moreno et al., 2008; Ruiz et al., 2010; Blanco et al., 2012). Regarding the cerebellum, the potentiating effects of cocaine on the GABA-mediated inhibition of Purkinje neurons were not observed after the selective depletion of noradrenergic neurons (Waterhouse et al., 1991). Moreover, cocaine-induced sensitization augmented the expression of cFos, jun-B, Homer 1b/c and Homer 3a/b in the cerebellar cortex (Couceyro et al., 1994; Jiménez-Rivera et al., 2000; Dietrich et al., 2007). These homer isoforms could be a crucial link between mGluR and IP_3_-dependent intracellular Ca^2+^ signaling, which are considered relevant steps for synaptic remodeling and long-term adaptive changes (Szumlinski et al., 2006). In addition, during the withdrawal period from repeated cocaine exposure, the levels of NR1 and NR2A-NMDA glutamate receptor subunits were reduced in rat cerebellum (Yamaguchi et al., 2002).

Classic studies (Giuffrida et al., 1999; Gorriti et al., 1999) established that both the blockade and desensitization of CB1 receptors facilitate psychostimulant actions. Recent studies (Mereu et al., 2013) indicate that the eCB signaling system is recruited at first cocaine administration to initiate plastic changes that lead to the onset of behavioral sensitization. These plastic changes have been shown in the prefrontocortical projections to basal ganglia neurons. However, there are no available data on the impact of cocaine combined with the induction of sensitization on the expression of the signaling machinery for endocannabinoids and glutamate. Because the interaction of catecholaminergic innervation on excitatory synapsis in the brain has been shown to be dependent on endocannabinoid signaling, we decided to investigate whether similar interactions occur in the cerebellum after repeated cocaine exposure. To this end, we investigated whether gene and protein expression of relevant components of the eCB, glutamate signaling system (receptors and synthesis/degradation enzymes) or the rate-limiting enzyme in catecholamine biosynthesis (tyrosine hydroxylase) were altered by acute cocaine administration. We also investigated whether these factors were altered after a cocaine sensitization (CS) regimen involving cocaine administration into the mouse cerebellum.

## MATERIALS AND METHODS

### ETHICS STATEMENT

The protocols for animal care and use were approved by the Ethics and Research Committee at the Hospital Carlos Haya and Universidad de Málaga. All experimental animal procedures were carried out in strict accordance with the European Communities directive 86/609/ECC (24 November 1986) and Spanish legislation (BOE 252/34367-91, 2005) regulating animal research. All efforts were made to minimize animal suffering and to reduce the number of animals used.

### ANIMALS AND HOUSING

We used male C57BL/6J mice (25 ± 5 *g*; Charles River Laboratories International, Wilmington, MA, USA) for behavioral procedures and gene and protein expression analyses. All animals were maintained at the vivarium of the University of Malaga. All animals were experimentally naïve and were housed in clear plastic cages in a temperature-controlled room (22 ± 2°C) with a 12 h light–dark cycle (lights on at 8:00 a.m.) with free access to Purina laboratory feed and tap water before the initiation of the experiments.

### DRUGS

Cocaine-HCl was obtained from Alkaliber S.A. (Madrid, Spain), dissolved in sterile saline (0.9% NaCl) just before experimentation, and administered intraperitoneally at doses of 10 (acute and priming) and 20 mg/kg (cocaine conditioning).

### APPARATUS AND GENERAL PROCEDURES

All mice were handled and habituated to the injection procedures once per day for 5 days prior to behavioral testing to reduce the effect of the non-specific stress of being handled on test behavior. All experiments were carried out between 08:00 and 20:00 h. The animals were acclimated to the experimental room for 30 min each day. Performance in the open field was recorded by a computed-based video tracking system (Smart v2.5®, Panlab, Barcelona, Spain). The maximum light intensity in the center of the open field was 100 lux. Four open fields (50 cm × 50 cm × 50 cm, Panlab) with gray backgrounds were used. The animals were placed in the center of the arena, and their behavior was recorded for 30 min. Horizontal locomotion was measured as total distance traveled (cm).

### ACUTE/REPEATED COCAINE ADMINISTRATION, CONDITIONED LOCOMOTION, AND COCAINE SENSITIZATION

Cocaine sensitization was conducted following a consecutive four-phase paradigm: cocaine conditioning, drug free period (resting), conditioned locomotion (CL) probe and CS or priming test. Firstly, two mouse groups were injected with cocaine (20 mg/kg body weight) or vehicle (0.9% NaCl) for five consecutive days and exposed to the open field (cocaine conditioning). For the next 5 days, all animals rested without the drug. Then, we evaluated the locomotor activity response induced by the association between repeated administration of cocaine and the location at which it exerted its stimulant effect by simulated administration with vehicle (CL response). On the last day, we assessed the presence of sensitization by lower-dose cocaine administration (priming: 10 mg/kg body weight). All animals were evaluated in the Open Field test to measure the distance traveled (cm) for 30 min with the video tracking system, except in the drug free period. There were four experimental groups (*n *= 8): animals that were conditioned with cocaine (20 mg/kg body weight) for 5 days (chronic pretreatment), rested for 5 days and were administered vehicle or cocaine (10 mg/kg body weight; acute treatment) and animals that were conditioned with vehicle for 5 days (chronic pretreatment), rested for 5 days, and then were treated with vehicle or cocaine (10 mg/kg body weight; acute treatment). Thus, the groups of animals were (*n *= 8): (1) chronic vehicle pretreatment and acute vehicle treatment (vehicle–vehicle group), (2) chronic vehicle pretreatment and acute cocaine treatment (vehicle–cocaine group), (3) chronic cocaine pretreatment and acute vehicle treatment (cocaine–vehicle group), and (4) chronic cocaine pretreatment and acute cocaine treatment (cocaine–cocaine group). Animals from these four groups were used for the behavioral studies and the gene and protein expression analyses.

### TISSUE COLLECTION

One hour after acute treatment with vehicle or cocaine, all animals were sacrificed by decapitation, and their brains were immediately dissected out, frozen on dry ice, and stored at -80°C. The brains were dissected in coronal brain slice sections (1 mm thick) on dry ice using razor blades and a mouse brain slicer matrix (Zivic Instruments). The cerebellar cortex was precisely removed with fine surgical instruments according to the Paxinos and Franklin (2004). Samples were stored at -80°C until they were used for gene and protein analyses.

### RNA ISOLATION AND QUANTITATIVE REAL-TIME PCR ANALYSIS

Real-time PCR was used to quantify the relative mRNA levels of the catecholamine synthesizing enzyme tyrosine hydroxylase (TH) and the relevant receptors and synthesis/degradation enzymes involved in eCB signaling [CB1 receptor, *N*-acyl phosphatidylethanolamine phospholipase D (NAPE-PLD), diacylglycerol lipase alpha/beta (DAGLα/β), monoacylglycerol lipase (MAGL) and fatty acid amino hydrolase (FAAH)] and glutamate signaling [liver-type glutaminase isoform (LGA), kidney-type glutaminase isoform (KGA), mGluR3/5 metabotropic receptors, and NR1/2A/2B/2C-NMDA and GluR1/2/3/4- (AMPA ionotropic receptor subunits)] in the cerebellum. Total RNA was isolated using the Trizol® method, according to the manufacturer’s instruction (Gibco BRL Life Technologies, Baltimore, MD, USA). The tissue was placed into 1 ml of Trizol Reagent (Invitrogen, Carlsbad, CA, USA) and homogenized with an IKA-Ultra-Turrax®T8 (IKA-Werke GmbH, Staufen, Germany). To ensure the purity of the mRNA sequences and exclude molecules smaller than 200 nucleotides, RNA samples were isolated with a RNeasy Minelute Cleanup Kit (Qiagen, Hilden, Germany), which included digestion with DNase I column (RNase-free DNase Set, Qiagen), according to the manufacturers’ instructions, and purified using RNeasy Mini Kit (Qiagen, Hilden, Germany). The total mRNA concentrations were quantified using a spectrophotometer (Nanodrop 1000 Spectrophotometer, Thermo Scientific, Rochester, NY, USA) to ensure A260/280 ratios of 1.8–2.0.

Reverse-transcription was carried out with 2 μg of mRNA using the Transcriptor Reverse Transcriptase kit and random hexamer primers (Transcriptor RT, Roche Diagnostic GmbH, Manheim, Germany). Negative controls included reverse-transcription reactions that omitted reverse transcriptase. The resultant cDNAs were used as templates for quantitative real-time PCR with an iCycler system (BioRad, Hercules, CA, USA) using the Quanti-Test SYBR Green PCR kit (Qiagen, Hilden, Germany). The primers used are described in **Table 1**. Oligonucleotides were provided by Sigma-Proligo (Proligo France SAS, Paris, France).

**Table 1:** Primers sequences used for RT-PCR^1^.

	Gene ID	GenBank accession numbers	Forward sense primers	Reverse antisense primers	Product size (bp)
	*Actb* (β-actin)	NM_007393	tacagcttcaccaccacagc	aaggaaggctggaagagagc	206

	*TH*	NM_009377.1	ccaaggaaagtgtcagagttgg	accctgcttgtattggaagg	150

eCB	*Cnr1* (CB1 receptor)	NM_007726.1	gctgcaatctgtttgctcag	ttgccatcttctgaggtgtg	201
	*Faah* (FAAH)	NM_010173.2	cggagagtgactgtgtggtg	tcagtgcctaaacccagagg	220
	*Napepld* (NAPE-PLD)	AB_112350	gcgccaagctatcagtatcc	tcagccatctgagcacattc	223
	*Mgll* (MAGL)	NM_011844.3	catggagctggggaacactg	ggagatggcaccgcccatggag	240
	*Dagla* (DAGLα)	NM_198114.1	agaatgtcaccctcggaatg	gcaggttgtaagtccgcaaa	153
	*Daglb* (DAGLβ)	NM_144915.2	aagcggccagatacattcac	ggataagcgacacgacaaag	246

*Glutamate sinthesizing*	*Gls2* (LGA)	NM_001033264	ttggaccatgcgctgcatcttg	gcactcggatcatgacgcctcac	190
enzymes	*Gls* (KGA)	NM_001081081	gcgagggcaaggagatggtg	ctctttcaacctgggatcagatgttc	179

Metabotropic glutamate	*Gmr3* (mGluR3)	NM_181850.2	tgagtggtttcgtggtcttg	tgcttgcagaggactgagaa	153
receptors	*Gmr5* (mGluR5)	BC096533.1	aagaaggagaaccccaacca	ttcggagactggagagtttg	179

NMDA ionotropic glutamate	*Grin1* (NR1)	NM_008169	gtgcaagtgggcatctacaa	tgggcttgacatacacgaag	157
receptor subunits	*Grin2a* (NR2A)	NM_008170	gtttgttggtgacggtgaga	aagaggtgctcccagatgaa	180
	*Grin2b* (NR2B)	NM_008171	atgtggattgggaggatagg	tcgggctttgaggatacttg	249
	*Grin2c* (NR2C)	NM_010350	ggaatggtatgatcggtgag	ccgtgaggcacattacaaac	225

AMPA ionotropic glutamate	*Gria1* (GluR1)	NM_008165.2	ttttctaggtgcggttgtgg	cctttggagaactgggaaca	210
receptor subunits	*Gria2* (GluR2)	NM_001039195.1	aaggaggaaagggaaacgag	ccgaagtggaaaactgaacc	217
	*Gria3* (GluR3)	NM_016886.2	caacaccaaccagaacacca	atcggcatcagtgggaaa	229
	*Gria4* (GluR4)	NM_019691.3	ttggaatgggatggtaggag	taggaacaagaccacgctga	250

1 Oligonucleotides were provided by Sigma-Proligo.

Quantification was carried out according to standard curves and was run simultaneously. The samples for each reaction were run in duplicate. The PCR product was separated by electrophoresis in a 1% agarose gel to verify fragment size and the absence of contaminating fragments, quantified by measuring the absorbance at 260 nm, and serially diluted to 10^-5^ pg/ml. Several 10-fold dilutions (10^-1^–10^-5^) were checked for optimal cycling on the iClycler system, and three of them were selected for the standard curves. Each reaction was run in duplicate and contained 2.5 μl of cDNA template, 8 μl of Master SYBR Green, 4.86 μl of PCR Ultra Pure Water and 0.64 μl of primers in a final reaction volume of 15 μl. Cycling parameters were 95°C for 15 min to activate DNA polymerase, then 30–40 cycles at 94°C for 15 s, temperature-specific annealing for each primer for 30 s and a final extension step of 72°C for 30 s, in which fluorescence was acquired. Melting curve analysis was performed to ensure that only a single product was amplified. Absolute values from each sample were normalized with regard to β-actin mRNA (constitutive gene), which was used as a reference standard. This internal standard was chosen based on a first analysis of a panel of housekeeping genes that included cyclophylin and transcription factor (specificity protein) 1.

### WESTERN BLOT ANALYSIS

Western blotting was used to quantify the relative protein levels of the eCB signaling system (CB1 receptor, NAPE-PLD, DAGLα/β, MAGL, and FAAH) in the cerebellum. Samples were homogenized in 50 mM Hepes buffer (pH 8) and 0.32 M sucrose buffer to obtain membrane protein extracts. The homogenate was centrifuged at 800 *g* for 10 min at 4°C, and the supernatant was centrifuged at 40000 *g* for 30 min. The pellets were resuspended in 50 mM Hepes buffer (pH 8) and pulverized using a homogenizer. Protein concentration was measured using the Bradford protein assay.

For immunoblotting, protein samples (40 μg) were separated on 10% (w/v) SDS-PAGE gels, transferred on to nitrocellulose membranes (BioRad) and controlled by Ponceau Red staining. After blocking with 5% (w/v) bovine serum albumin (BSA) in PBST buffer (0.1% Tween 20 in PBS) at room temperature for 1 h, membranes were incubated with the primary antibodies overnight at 4°C, as was described previously (Suarez et al., 2008): anti-CB1 receptor (Cayman, cat. no. 101500) diluted 1:200, anti-DAGLα (produced in our laboratory) diluted 1:100, anti-DAGLβ (produced in our laboratory) diluted 1:100, anti-NAPE-PLD (produced in our laboratory) diluted 1:100, anti-FAAH (Cayman, cat. no. 101600) diluted 1:100 and anti-MAGL (Cayman, cat. no. 100035) diluted 1:200. After incubation with a peroxidise-conjugated goat anti-rabbit IgG (H + L) antibody (Promega) diluted 1:2500 for 1 h at room temperature, the membranes were revealed by the Western Blotting Luminol Reagent kit (Santa Cruz Biotechnology). Specific protein bands were visualized and quantified by chemiluminescence using an imaging AutoChemi^TM^ UVP BioImagin System (LTF Labortechnik). β-actin was quantified and used as a loading control (anti-β-actin, Sigma, cat. no. A5316, diluted 1:1000).

### STATISTICAL ANALYSIS

Data are expressed as the mean ± standard error of the mean (SEM) for at least eight determinations per experimental group. Statistical significance for behavioral data was assessed by one-way/repeated measures analysis of variance (ANOVA) and a *post hoc* Newman–Keuls test. Statistical significance of gene and protein quantification was obtained by two-way ANOVA with the two factors being chronic pretreatment (conditioning with vehicle or cocaine for 5 days) and acute treatment (vehicle or cocaine for 1 day), followed by Bonferroni test as a priori non-orthogonal contrast test. *P *< 0.05 was considered statistically significant.

## RESULTS

### COCAINE-ASSOCIATED BEHAVIORS

**FIGURE 1:**
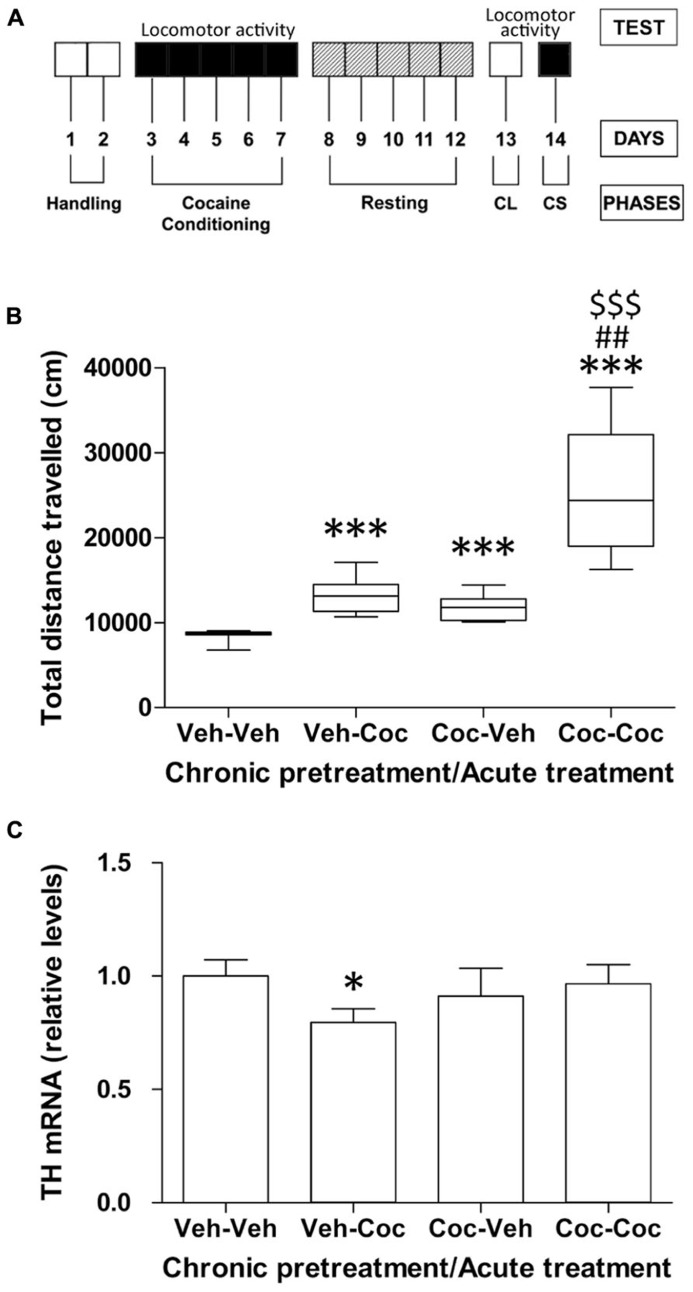
**(A)** Schematic representation of the different phases of cocaine behavior such as acute cocaine administration (10 mg/kg), cocaine conditioning (20 mg/kg), resting, conditioned locomotion (CL) and cocaine sensitization (CS, 10 mg/kg). **(B)** Effect of acute cocaine administration (chronic vehicle + acute cocaine), CL (chronic cocaine + acute vehicle) and cocaine sensitization (chronic cocaine + acute cocaine) on distance traveled for 30 min measured in the Open Field. Box-and-whisker diagram represents the statistical distribution of the data (*n *= 8/group). Newman–Keuls: ^***^P < 0.001 *vs.* vehicle–vehicle group, ^##^*P *< 0.01 *vs.* vehicle–cocaine group, ^$$$^*P *< 0.001 *vs.* cocaine–vehicle group. **(C)** Relative mRNA levels of the catecholamine synthesizing enzyme tyrosine hydroxylase (TH) in the mouse cerebellum after chronic pretreatment (vehicle and cocaine) and acute treatment (vehicle and cocaine). Histograms represent the mean ± SEM (*n *= 8/group). Two-way ANOVA: ns, no significance. Bonferroni: ^*^P < 0.05 *vs.* vehicle–vehicle group.

**Figure 1A** shows a schematic representation of the schedule used for analysis of cocaine-associated behaviors. Briefly, it included the analysis of locomotion in the open field during repeated cocaine administration (5 days-cocaine conditioning), a 5 days-resting period, 1 day for testing CL and 1 day for testing CS (priming). Mice pretreated with vehicle for 5 days, rested and then treated with vehicle (vehicle–vehicle group) were used as the baseline for locomotion (**Figure 1B**). Mice pretreated with vehicle and then treated with one administration of cocaine (10 mg/kg; vehicle–cocaine group) showed a significant increase in locomotor activity (^***^P < 0.001) compared with the vehicle–vehicle group. After 5 days of cocaine conditioning (20 mg/kg) and 5 days of resting, animals were then subjected to an administration of vehicle or cocaine (10 mg/kg). Repeated cocaine-conditioned animals receiving vehicle (cocaine–vehicle group) showed an increase in locomotion, reflecting the acquisition of a CL response (^***^P < 0.001) compared with the vehicle–vehicle group (non-conditioned control group). This effect was produced by association between the features of the environment and the rewarding properties of cocaine after 5 days of injections. Repeated cocaine-conditioned animals receiving cocaine (cocaine–cocaine group) showed a significant CS response as a consequence of significantly higher locomotor activity compared with the conditioning vehicle groups (vehicle–vehicle, ^***^P < 0.001 and vehicle–cocaine, ^##^*P *< 0.01). Moreover, CS-associated locomotor activity (cocaine–cocaine group) was significantly higher (^$$$^*P *< 0.001) than CL (cocaine–vehicle group; **Figure 1B**).

### GENE EXPRESSION OF CATECHOLAMINE BIOSYNTHESIS

To address whether cocaine treatment was associated with alterations in the state of the catecholaminergic system, we analyzed the gene expression of TH, the rate-limiting enzyme in catecholamine biosynthesis, in the cerebellum of the vehicle–vehicle (V–V), vehicle–cocaine (V–C), cocaine–vehicle (C–V), and cocaine–cocaine (C-C) mice. Acute cocaine exposure (vehicle–cocaine group) induced a decrease in TH gene expression (^*^P < 0.05) compared with the vehicle–vehicle group (**Figure 1C**). Repeated cocaine-conditioned animals receiving vehicle (cocaine–vehicle group) or cocaine (cocaine–cocaine group) did not show any effect on TH expression derived from acute treatment, chronic pre-treatment or the interaction between these factors.

### GENE AND PROTEIN EXPRESSION OF eCB SIGNALING COMPONENTS

To address the possible neuroadaptive changes in relevant components of eCB signaling associated with the effects of acute cocaine exposure and cocaine conditioning or sensitization, we analyzed the gene and protein expression of CB1 receptors and the enzymes of eCB synthesis (DAGLα/β and NAPE-PLD) and degradation (FAAH and MAGL) in the cerebellum of vehicle–vehicle (V–V), vehicle–cocaine (V–C), cocaine–vehicle (C–V), and cocaine–cocaine (C–C) mice. To analyze whether the differential expression of either eCB producing or degrading enzymes can result in altered eCB tone in the cerebellum in the four experimental groups, we also calculated the ratios between NAPE-PLD and FAAH expression and between DAGLα/β and MAGL expression. These ratios can suggest possible changes in *N*-acylethanolamines (NAEs) and 2-arachidonylglycerol (2-AG) levels, respectively.

Western blot analysis showed that antibodies used against the components of the eCB signaling system revealed bands with expected molecular weights in the cerebellum, as was previously described (Suarez et al., 2008). CB1 immunoblotting revealed a prominent band at approximately 60 kD, NAPE-PLD at 46 kD, FAAH at 63 kD, DAGLα at 120 kD, DAGLβ at 76 kD, and MAGL at 35–37 kD.

#### eCB mRNA levels in the cerebellum

**FIGURE 2:**
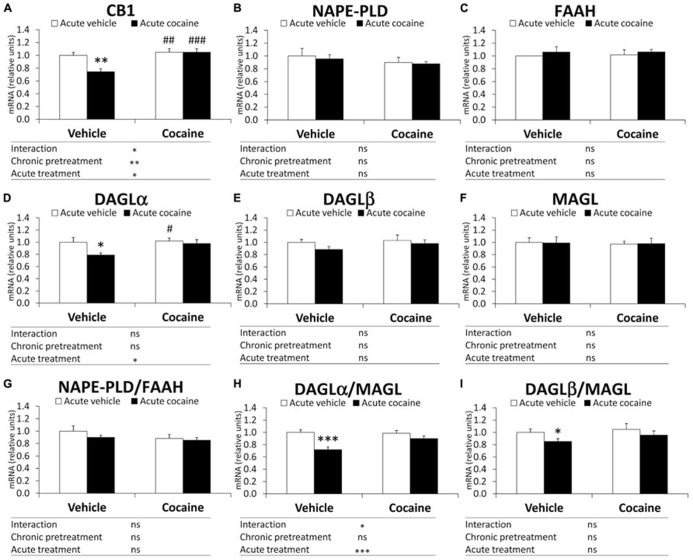
**Relative mRNA levels of components of the eCB signaling system [CB1 (A), NAPE-PLD (B), FAAH (C), DAGLα (D), DAGLβ (E), MAGL (F), NAPE-PLD (G), DAGLα/MAGL (H), and DAGLβ/MAGL (I)] in the mouse cerebellum after chronic pretreatment (vehicle and cocaine) and acute treatment (vehicle and cocaine)**. Histograms represent the mean ± SEM (*n *= 8/group). Two-way ANOVA: ns, no significance; ^*^P < 0.05, ^**^P < 0.01, ^***^P < 0.001. Bonferroni: ^*^P < 0.05, ^**^P < 0.01, ^***^P < 0.001 *vs.* vehicle–vehicle group; ^#^*P *< 0.05, ^##^*P *< 0.01, ^###^*P *< 0.001 *vs.* vehicle–cocaine group.

Two-way ANOVA analysis only showed a chronic pretreatment effect in CB1 gene expression (*F*_1,27_ = 10.73, *P *= 0.0029; **Figure 2A**). Significant effects of acute treatment were also found in the cerebellar gene expression of CB1 and DAGLα and the DAGLα/MAGL ratio (CB1: *F*_1,27_ = 5.66, *P *= 0.024; DAGLα: *F*_1,27_ = 4.44, *P *= 0.044; DAGLα/MAGL: *F*_1,27_ = 16.52, *P *= 0.0004; **Figures 2A,D,H**). Interaction between factors was detected in the gene expression of CB1 and the DAGLα/MAGL ratio (CB1: *F*_1,27_ = 5.66, *P *= 0.024; DAGLα/MAGL: *F*_1,27_ = 4.57, *P *= 0.041; **Figures 2A,H**), indicating that acute treatment with cocaine did not have the same effect on the gene expression of CB1 and the DAGLα/MAGL ratio after chronic pretreatment. Bonferroni analysis showed that acute cocaine (vehicle–cocaine group) induced a decrease in CB1 gene expression (^**^P < 0.01), DAGLα (^*^P < 0.05) and the DAGLα-β/MAGL ratio (^***^P < 0.001 and ^*^P < 0.05, respectively), but not in gene expression of NAPE-PLD, FAAH, DAGLβ and MAGL or the NAPE-PLD/FAAH ratio (**Figure 2**). Repeated cocaine-conditioned animals receiving vehicle (cocaine–vehicle group) or cocaine (cocaine–cocaine group) showed no effect on gene expression in the eCB signaling components analyzed compared with the vehicle–vehicle group. However, the cerebella of the repeated cocaine-conditioned animals receiving vehicle presented increased CB1 and DAGLα mRNA levels (^##^*P *< 0.01 and ^#^*P *< 0.05, respectively), whereas repeated cocaine-conditioned animals receiving cocaine only showed an increase in CB1 mRNA levels (^###^*P *< 0.001) compared with the vehicle–cocaine group (**Figures 2A,D)**.

#### eCB protein levels in the cerebellum

**FIGURE 3:**
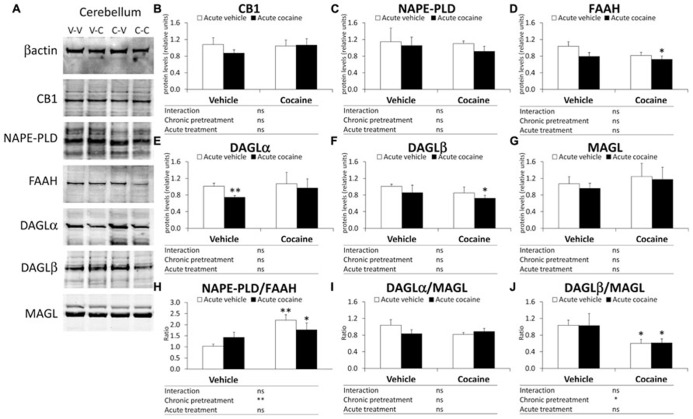
**Representative immunoblots (A) and relative protein levels of components of the eCB signaling system [CB1 (B), NAPE-PLD (C), FAAH (D), DAGLα (E), DAGLβ (F), MAGL (G), NAPE-PLD (H), DAGLα/MAGL (I), and DAGLβ/MAGL (J)] in the mouse cerebellum after chronic pretreatment (vehicle and cocaine) and acute treatment (vehicle and cocaine)**. Histograms represent the mean ± SEM (*n *= 8/group). Two-way ANOVA: ns, no significance; ^*^P < 0.05, ^**^P < 0.01. Bonferroni: ^*^P < 0.05, ^**^P < 0.01 *vs.* vehicle–vehicle group.

**Figure 3A** illustrates representative immunoblots showing protein expression of the eCB components analyzed in the cerebellum of the four experimental groups (V–V, V–C, C–V, and C–C). Two-way ANOVA analysis showed a chronic pretreatment effect only in the NAPE-PLD/FAAH ratio (*F*_1,12_**= 10.31, *P *= 0.0075) and DAGLβ/MAGL ratio (*F*_1,12_ = 6.02, *P *= 0.0304; **Figures 3H,J**), but not in the remaining eCB components. A significant effect of acute treatment was not observed in the cerebellar protein expression of the eCB components analyzed. Interaction between factors was not detected, indicating that acute cocaine treatment had the same effect on the protein expression of the eCB components as chronic pretreatment. Bonferroni analysis showed that acute cocaine (vehicle–cocaine group) induced a decrease in the gene expression of DAGLα, but not in the gene expression of the remaining eCB components and ratios analyzed (**Figure 3**). The cocaine–vehicle group showed an increase in the NAPE-PLD/FAAH ratio (^**^P < 0.01) and a decrease in the DAGLβ/MAGL (^*^P < 0.05) ratio compared with the vehicle–vehicle group (**Figures 3H,J**). The cocaine–cocaine group showed lower protein expression of FAAH and DAGLβ, a lower DAGLβ/MAGL ratio and a higher NAPE-PLD ratio compared with the vehicle–vehicle group (^*^P < 0.05 in all cases; **Figures 3D,F,H,J**).

### GENE EXPRESSION OF GLUTAMATE SIGNALING COMPONENTS

To address whether the cocaine behavior-related changes observed in eCB signaling components were associated with an alteration in the glutamatergic state, we analyzed the gene expression of the glutamate synthesizing enzymes LGA and KGA, mGluR3/5 metabotropic receptors, and NR1/2A/2B/2C-NMDA and GluR1/2/3/4-AMPA ionotropic receptor subunits in the cerebellum of the vehicle–vehicle (V–V), vehicle–cocaine (V–C), cocaine–vehicle (C–V), and cocaine–cocaine (C–C) mice.

**FIGURE 4:**
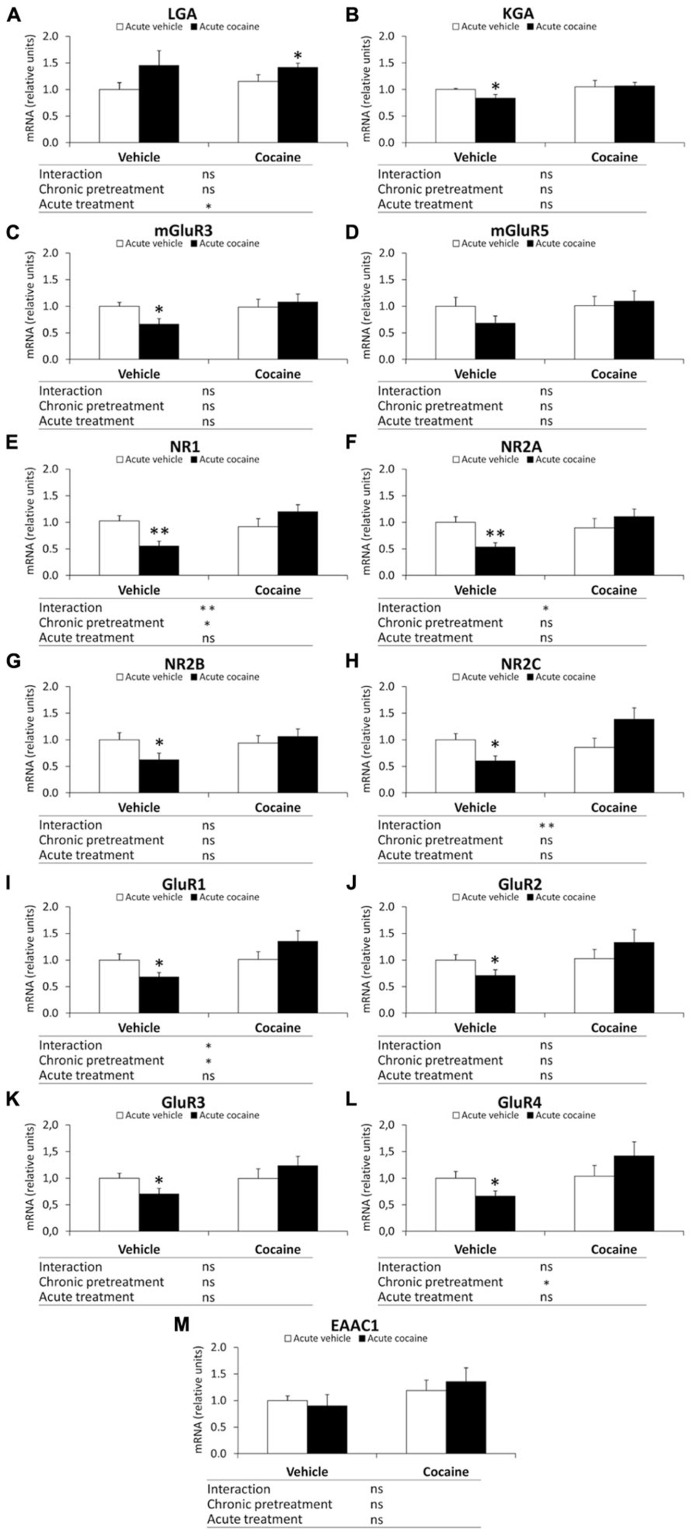
**Relative mRNA levels of components of the glutamate signaling system [LGA (A), KGA (B), mGluR3/5 (C,D), NR1/2A/2B/2C (E–H), and GluR1/2/3/4 (I–L) and EAAC1 (M)] in the mouse cerebellum after chronic pretreatment (vehicle and cocaine) and acute treatment (vehicle and cocaine)**. Histograms represent the mean ± SEM (*n *= 8/group). Two-way ANOVA: ns, no significance; ^*^P < 0.05, ^**^P < 0.01. Bonferroni: ^*^P < 0.05, ^**^P < 0.01 *vs.* vehicle–vehicle group.

Two-way ANOVA analysis showed a chronic pretreatment effect in the gene expression of NR1, GluR1 and GluR4 (NR1: *F*_1,28_ = 4.60, *P *= 0.04; GluR1: *F*_1,28_ = 5.49, *P *= 0.026; GluR4: *F*_1,28_ = 4.53, *P *= 0.042; **Figures 4E,I,L**). Significant effects of the acute treatment were only observed in the cerebellar gene expression of LGA (*F*_1,28_ = 4.30, *P *= 0.047; **Figure 4A**). Interaction between factors was also found in the NR1, NR2A, NR2C and GluR1 mRNA levels (NR1: *F*_1,28_ = 9.17, *P *= 0.0052; NR2A: *F*_1,28_ = 6.31, *P *= 0.018; NR2C: *F*_1,28_ = 8.46, *P *= 0.007; GluR1: *F*_1,28_ = 5.20, *P *= 0.0304; **Figures 4E,F,H,I**), indicating that acute treatment of cocaine differentially affects gene expression of NR1, NR2A, NR2C and GluR1 in a chronic pretreatment-dependent manner. Bonferroni analysis indicated that acute cocaine exposure (vehicle–cocaine group) induced a decrease in the gene expression of most glutamate signaling components analyzed, such as KGA (^*^P < 0.05), mGluR3 (^*^P < 0.05), NR1 (^**^P < 0.01), NR2A (^**^P < 0.01), NR2B (^*^P < 0.05), NR2C (^*^P < 0.05), GluR1(^*^P < 0.05), GluR2 (^*^P < 0.05), GluR3 (^*^P < 0.05), and GluR4 (^*^P < 0.05), compared with the vehicle–vehicle group (**Figure 4**). Repeated cocaine-conditioned animals receiving vehicle (cocaine–vehicle group) did not show any significant change in the gene expression of the glutamate components analyzed. Repeated cocaine-conditioned animals receiving cocaine (cocaine–cocaine group) only showed a significant increase in LGA mRNA levels compared with the vehicle–vehicle group (^*^P < 0.05; **Figure 4A**).

## DISCUSSION

The present study confirms that the eCB signaling system is a modulatory system that differentially modifies the expression of its components as a result of acute versus repeated cocaine exposure in the cerebellum. Overall, the data suggest that, in this central nervous system structure, acute cocaine exposure reduced the expression of the 2-AG-synthesizing enzyme DAGLα. As a consequence, the ratio of DAGLα/MAGL dropped, suggesting reduced production of the endocannabinoid 2-AG, which is responsible for inhibiting the excitatory glutamatergic inputs of granule cells because DAGLα is expressed in Purkinje dendrites (Suarez et al., 2008). No changes were observed in the machinery that produces anandamide (AEA), the second major endocannabinoid. Repeated administration of cocaine resulted in a differential response in the 2-AG and AEA turnover machinery. Data associated with CS after chronic cocaine administration indicated decreased protein expression of FAAH and DAGLβ [production in Purkinje dendrites and cell bodies described by Suarez et al. (2008)], suggesting opposite availabilities for *N*-acylethanolamides (NAEs) and 2-AG, respectively. The lack of eCB-mediated inhibition might be partially responsible for the well-described hyperglutamatergic state associated with prolonged cocaine action (Cornish and Kalivas, 2000). Thus, after chronic cocaine exposure, we observed an activated glutamatergic state in the cerebellum as a consequence of increased expression of the glutamate synthetizing enzyme LGA and normalized expression of all metabotropic and ionotropic glutamate receptors. In contrast, after acute cocaine exposure, we observed a down-regulation of the expression of the glutamate synthetizing enzyme KGA, which can reflect a hypoactivated glutamatergic state in the cerebellum. This hypothetic hypoglutamatergic state was associated with decreased expression of key signaling receptors such as the metabotropic mGluR3 receptor and all subunits of both ionotropic (NMDA and AMPA) glutamate receptors. Because cerebellar circuits are involved in the control of motor planning, motor learning, execution and coordination, an intact endogenous cannabinoid system is required to regulate sensorimotor integration. Our hypothesis is as follows: (1) An excessive deficit of 2-AG production after acute cocaine exposure may facilitate glutamate-mediated activation of Purkinje inhibitory GABAergic transmission, leading to a compensatory hypoglutamatergic (biosynthesis of the enzyme KGA and glutamate receptors) state in the cerebellum. This dysregulation of both the eCB and glutamate signaling systems can result in disrupted control of action and coordination, especially in sensorimotor integration. (2) The decreased expression of the eCB-degradation FAAH and eCB-producer DAGLβ, which suggest opposite tones for NAEs and 2-AG, respectively, could be explained by a neuroadaptation of the eCB signaling system to chronic cocaine exposure that finally leads to a hyperactivated glutamatergic state (biosynthesis enzyme LGA) in the cerebellum (see **Table 2** for summary).

**Table 2:** Summary of the main effects observed on the endocannabinoid and glutamate systems after acute cocaine, conditioning locomotion, and cocaine sensitization^2^.

Endocannabinoid system	Acute			Conditioning locomotion		Cocaine sensitization	
**mRNA/protein levels**
CB1	↓^**^	–	↑##	–	↑##	–
NAPE-PLD	–	–	–	–	–	–
FAAH	–	–	–	–	–	↓^*^
DAGLα	↓^*^	↓^*^	↑#	–	–	–
DAGLβ	–	–	–	–	–	↓^*^
MAGL	–	–	–	–	–	–
NAPE-PLD/FAAH	–	–	–	↑^**^	–	↑^*^
DAGLα/MAGL	↓^***^	–	–	–	–	–
DAGLβ/MAGL	↓^*^	–	–	↓^*^	–	↓^*^
**Glutamatergic system**
** mRNA levels**
LGA	–		–		↑^*^
KGA	↓^*^		–		–
mGluR3	↓^*^		–		–
mGluR5	–		–		–
NR1	↓^**^		–		–
NR2A	↓^**^		–		–
NR2B	↓^*^		–		–
NR2C	↓^*^		–		–
GluR1	↓^*^		–		–
GluR2	↓^*^		–		–
GluR3	↓^*^		–		–
GluR4	↓ ^*^		–		–
EAAC1	–		–		–
TH	↓^*^		–		–

1 *P < 0.05, ^**^P < 0.01, ^***^P < 0.001 *vs.* vehicle–vehicle group; ^#^*P *< 0.05, ^##^*P *< 0.01 *vs.* vehicle–cocaine group.

Analysis of the expression of the synthesis pathways for eCB revealed that DAGLα was reduced in the cerebellum of acute cocaine-exposed animals. The trend of the relative synthetizing/degrading enzymes suggested a decrease in 2-AG and no change in AEA. Because acute first exposure to cocaine increased eCB formation, most likely via the activation of DA D2 receptors in the striatum (Giuffrida et al., 1999; Centonze et al., 2004), we hypothesized that, in the cerebellum, acute administration of cocaine might lead to a hyposensitization of the noradrenergic-driven (most likely beta-receptor mediated) eCB inhibitory signal and, as a consequence, a hyposensitization of the eCB-driven glutamatergic excitatory signal. There is strong evidence that eCBs modulate noradrenergic signaling (Kirilly et al., 2013). This signaling effect can be addressed by adapting the machinery needed for 2-AG (DAGLα) and glutamate (KGA) production.

Regarding the adaptations observed in the eCB signaling system, acute cocaine caused a decrease in CB1 mRNA levels but, after chronic cocaine exposure, CB1 gene expression returned to initial levels (compared with the vehicle–vehicle group). Compared with the vehicle–cocaine group, prolonged exposure produced an increase in the CB1 gene (but not protein) expression in the cerebellum, which can be mainly associated with an increase in the NAPE-PLD/FAAH ratio and a decrease in the DAGLβ/MAGL ratio (observed at the protein level). This combination is suggestive of an increased availability of NAEs and a reduced availability of 2-AG, respectively, which may be induced by CS after chronic cocaine administration. Although a previous report indicated that enhanced bioavailability of AEA, derived from inhibition of either AEA uptake or AEA degradation, attenuated behavioral responses to cocaine (Chaperon et al., 1998; Fattore et al., 1999; Adamczyk et al., 2009), our results suggest that in the absence of external pharmacological manipulation of eCB production, endocannabinoid neuroadaptations are insufficient to compensate for the overactivity derived from chronic cocaine treatments. Thus, the up-regulation of CB1 gene expression (most likely associated with the local circuitry of the cerebellar cortex) and the increased availability of AEA cannot counteract glutamatergic overactivity. The decrease in 2-AG, which is the main retrograde signal, is not sufficiently compensated by enhanced AEA. Supporting this finding, a recent report by Orio and co-workers (Orio et al., 2009) showed that AEA and 2-AG release, as measured by microdialysis, was reduced in rats self-administering cocaine. This finding was associated with enhanced expression of CB1 receptors. In a previous study by our group (Rivera et al., 2013), we demonstrated a similar finding in the Hc of Lewis rats self-administering cocaine. Moreover, we cannot ignore the possibility that the observed changes in NAPE-PLD/FAAH may involve non-CB1-acting endocannabinoids, such as oleoylethanolamide (OEA). This interpretation arises because beta-adrenergic activation stimulates synthesis of OEA in response to cold exposure in adipocytes and to satiety in the small intestine (LoVerme et al., 2006; Fu et al., 2011). Moreover, OEA has been recently proposed to control cocaine-associated behaviors (Bilbao et al., 2013). Concerning the CB2 receptor, although we have not measured its expression in the present study, we cannot rule out a role for this receptor in the neuroadaptive responses to cocaine, as recently suggested (Xi et al., 2011; Aracil-Fernandez et al., 2012).

Glutamate seems to be a primary mediator of cocaine-induced behaviors associated with drug-seeking (Cornish and Kalivas, 2000). For instance, stimulation of glutamate receptors in the NAc augments the reinforcing effect of cocaine, and increased glutamatergic neurotransmission may be involved in facilitating relapse to cocaine-seeking behavior (Cornish et al., 1999). Activation of group I (mGluR1/5) metabotropic receptors resulted in an increase in NAc extracellular glutamate levels (Swanson et al., 2001). Moreover, stimulation of mGluR5 in the PFCx is sufficient to induce CS (Timmer and Steketee, 2012), whereas the administration of mGluR5 antagonists attenuated cocaine priming- and cue-induced reinstatement of cocaine seeking (Platt et al., 2008; Kumaresan et al., 2009). Our present data agree with these findings, suggesting the induction of glutamate synthesis (increased LGA gene expression) in CS after chronic cocaine exposure. However, the increase in the glutamate synthesizing enzyme LGA is not associated with an enhancement of the expression of any metabotropic or ionotropic glutamate receptor analyzed as a compensatory mechanism to counteract the putative hyperactivation of these receptors.

In summary, acute and chronic cocaine exposure differentially modulates the expression of the eCB and glutamate signaling machinery in the cerebellar cortex. After acute cocaine exposure, the changes are compatible with a situation in which the reduced inhibitory actions of 2-AG on glutamatergic terminals cause decreased synthesis of catecholamine and glutamate. After repeated cocaine exposure, increased synthesis of glutamate results from dysregulation of eCB availability (increased NAEs and decreased 2-AG) in the cerebellum. This situation can contribute to the induction of behavioral sensitization, a process thought to be relevant for psychostimulant addiction.

## Conflict of Interest Statement

The authors declare that the research was conducted in the absence of any commercial or financial relationships that could be construed as a potential conflict of interest.

## Abbreviations

2-AG: 2-arachidonylglycerol
AEA: anandamide
CB1: cannabinoid receptor type 1
CL: conditioned locomotion
CPP: conditioned place preference
CS: cocaine sensitization;
DA: dopamine
DAGLα/β: diacylglycerol lipase alpha/beta
eCBs: endocannabinoids
FAAH: fatty acid amino hydrolase
GluR1/2/3/4: AMPA-ionotropic glutamatergic receptor
Hc: hippocampus
KGA: kidney-type glutaminase isoform
LGA: liver-type glutaminase isoform
MAGL: monoacylglycerol lipase
mGluR: metabotropic glutamatergic receptor
NAc: nucleus accumbens
NAE: *N*-acylethanolamine
NAPE-PLD: *N*-acyl phosphatidylethanolamine phospholipase
D NR1/2A/2B/2C: NMDA-ionotropic glutamatergic receptor
PFCx: prefrontal cortex
RT-qPCR: reverse-transcription real-time quantitative-polymerase chain reaction
Str: dorsal striatum
